# Design Considerations for UTI Digital Solutions for People Living with Dementia: Insights from Caregivers

**DOI:** 10.1093/geroni/igaf122.2575

**Published:** 2025-12-31

**Authors:** Kuan-Ching Wu, Oleg Zaslavsky

**Affiliations:** Emory University, Atlanta, Georgia, United States; University of Washington, Seattle, Washington, United States

## Abstract

Urinary tract infections (UTIs) are a leading cause of hospitalization and emergency room visits among people living with dementia (PLWD). Dementia caregivers often struggle to recognize early UTI symptoms and access timely guidance, increasing the risk of complications. This study aims to prototype Goodbye UTI (GUTI), a digital solution designed to support caregivers in UTI prevention and management. Following the framework of the four-steps human-centered design (HCD) approach, we identified key design considerations for GUTI. Semi-structured interviews were conducted with 11 dementia caregivers (aged 42–82, M = 68.74, SD = 11.41) from December 2023 to June 2024 to explore their needs and preferences for UTI digital solutions. Data were analyzed using inductive thematic coding with ATLAS.ti to extract insights on user needs, requirements, and context of use. Caregivers reported challenges such as emotional strain, communication difficulties, limited knowledge, and the need for accessible health information. They emphasized the importance of a simple interface with symptom tracking, reminders, and educational resources. Based on these insights, we developed a functional matrix with eight core design considerations and created ten prototype screenshots in Figma demonstrating key features. Findings highlight the need for intuitive, personalized, and actionable digital tools for UTI management in dementia care. GUTI addresses these needs by prioritizing reminder, real-time guidance, and caregiver support. Future research should focus on iterative design in prototype testing and development, usability and the effectiveness of GUTI in real-world caregiving context.

